# Sex-specific involvement of the Notch–JAG pathway in social recognition

**DOI:** 10.1038/s41398-022-01867-4

**Published:** 2022-03-10

**Authors:** Hanna Jaaro-Peled, Melissa A. Landek-Salgado, Nicola G. Cascella, Frederick C. Nucifora, Jennifer M. Coughlin, Gerald Nestadt, Thomas W. Sedlak, Joelle Lavoie, Sarah De Silva, Somin Lee, Katsunori Tajinda, Hideki Hiyama, Koko Ishizuka, Kun Yang, Akira Sawa

**Affiliations:** 1 Department of Psychiatry, Johns Hopkins University School of Medicine, Baltimore, MD 21205 USA; 2 Department of Neuroscience, Johns Hopkins University School of Medicine, Baltimore, MD 21205 USA; 3 Department of Biomedical Engineering, Johns Hopkins University School of Medicine, Baltimore, MD 21205 USA; 4 Department of Genetic Medicine, Johns Hopkins University School of Medicine, Baltimore, MD 21205 USA; 5 Department of Mental Health, Johns Hopkins University Bloomberg School of Public Health, Baltimore, MD 21205 USA

**Keywords:** Neuroscience, Psychology

## Abstract

Under the hypothesis that olfactory neural epithelium gene expression profiles may be useful to look for disease-relevant neuronal signatures, we examined microarray gene expression in olfactory neuronal cells and underscored Notch–JAG pathway molecules in association with schizophrenia (SZ). The microarray profiling study underscored *JAG1* as the most promising candidate. Combined with further validation with real-time PCR, downregulation of *NOTCH1* was statistically significant. Accordingly, we reverse-translated the significant finding from a surrogate tissue for neurons, and studied the behavioral profile of *Notch1*^*+/*−^ mice. We found a specific impairment in social novelty recognition, whereas other behaviors, such as sociability, novel object recognition and olfaction of social odors, were normal. This social novelty recognition deficit was male-specific and was rescued by rapamycin treatment. Based on the results from the animal model, we next tested whether patients with psychosis might have male-specific alterations in social cognition in association with the expression of *NOTCH1* or *JAG1*. In our first episode psychosis cohort, we observed a specific correlation between the expression of *JAG1* and a face processing measure only in male patients. The expression of *JAG1* was not correlated with any other cognitive and symptomatic scales in all subjects. Together, although we acknowledge the pioneering and exploratory nature, the present work that combines both human and animal studies in a reciprocal manner suggests a novel role for the Notch–JAG pathway in a behavioral dimension(s) related to social cognition in psychotic disorders in a male-specific manner.

## Introduction

Through the advancement of psychiatric genetics, many biological pathways have been underscored in association with neuropsychiatric disorders [1–6]. Some genes that were originally highlighted in studies with specific pedigrees may not be well reproduced as major risk factors in genome wide association studies (GWASs), which include genes for Notch signaling, in particular NOTCH4, for schizophrenia (SZ) [7–13]. As a complementary approach to look for the molecular drivers of the pathophysiology of neuropsychiatric disorders, molecular expression studies using tissue from patients and healthy subjects are well appreciated [14–18]. Although expression levels of key molecular drivers directly impact neurobiology, they are regulated not only by *cis*-elements (which can be easily identified by genetic studies) but also by *trans*-elements (which may not be identified by genetic studies). Thus, expression studies can complement the possible limitation associated with missing heritability or provide key molecules underlying pathophysiology.

The Notch pathway is one of the major cell–cell-signaling pathways regulating cell differentiation and development [19]. Mammals have four transmembrane Notch receptors (Notch1–4) that bind two classes of ligands: Jagged (Jag1 and Jag2) and Delta-like (Dll1, Dll3, and Dll4). Upon ligand binding, Notch undergoes proteolytic cleavage releasing the Notch Intracellular Domain (NICD), which then translocates to the cell nucleus to modify gene expression. In the developing brain, Notch activation inhibits neurogenesis, maintains the neural progenitor state, and affects binary fate choices. The Notch pathway has mainly been studied in the context of brain development, but may also play a role in the adult brain [20].

Olfactory neural epithelium is easily accessible in living people via nasal biopsy, and multiple research groups have utilized this surrogate tissue to obtain molecular signatures relevant to the brain [16, 21–23]. Using laser-captured microdissection, our team has enriched neurons from the biopsied tissue [15, 24, 25]. In the present study, we hypothesized that olfactory neural epithelium gene expression profiles might be useful to look for disease-relevant neuronal signatures, in particular obtaining possible molecular leads to initiate a biological study for neuropsychiatric disorders. Accordingly, we examined microarray gene expression in the neural tissue and underscored Notch–JAG pathway molecules in association with SZ. After finding downregulation of the Notch–JAG pathway molecules, we studied *Notch1* heterozygous knockout mice and discovered a male-specific impairment in social novelty recognition. Lastly, we returned to humans to examine the sex-specific association of NOTCH1 or JAG1 with clinical features, particularly paying attention to a behavioral dimension in social cognition.

## Materials/subjects and methods

### Microarray study of olfactory neural epithelium from SZ patients and healthy controls

#### The cohort of chronic SZ

The study with the chronic SZ cohort was approved by the Johns Hopkins School of Medicine Institutional Review Board and performed in accordance with the Code of Ethics of the World Medical Association. We obtained written informed consent from adult participants aged 18 years or older. Patients with chronic SZ were diagnosed based on the DSM-IV [26] by a board certified psychiatrist (NGC), and the recruitment chart was also reviewed by other psychiatrists (FCN, JMC, GN, TWS, and AS). The average duration of illness of patients is 18.72 years (SD = 10.78 years). Through this team approach, we constantly monitored the inter-rater reliability in any assessment. The patients were recruited from outpatient units in the Johns Hopkins Schizophrenia Center. Healthy controls were recruited from the general population through flyers posted at Johns Hopkins Medicine and an ad hoc advertisement in a local magazine. The inclusion and exclusion criteria for all study participants were: (1) no history of traumatic brain injury with loss of consciousness for >1 h; (2) no history of drug abuse within 6 months of the study; (3) no history of drug dependence within 12 months of the study, and (4) no history of untreated major medical illnesses. We used 18 SZ patients and 18 healthy controls for the molecular expression study (measured by microarray). There is no genomic information, which is a limitation of this cohort.

#### Microarray for the expression study of the chronic SZ cohort

We have previously published the method for the microarray study that was also employed in the present study [15, 25]. In short, olfactory neural epithelium was obtained by nasal biopsy from patients with chronic SZ and healthy controls. Neuronal epithelium was enriched by laser capture microscopy. The microarray study was performed using Affymetrix U133 Plus2.0. Data analysis was performed using the Partek Genomics Suite software (version 6.5, Partek) and R (http://www.rproject.org/, version 3.1.1) with Bioconductor packages (http://www.bioconductor.org/). Raw intensities were normalized using the GC-robust multi-array average. For differential gene expression analysis, one-way analysis of variance (ANOVA) was used to test the mean differences between two groups. The ANOVA *p*-values were adjusted using the Benjamini–Hochberg procedure to control the false discovery rate (FDR). The raw data are deposited in the Gene Expression Omnibus archive at the National Center for Biotechnology Information

(GSE73129: http://www.ncbi.nlm.nih.gov/geo/query/acc.cgi?acc = GSE73129).

#### Real-time quantitative PCR

Gene expression was quantified using real-time quantitative polymerase chain reaction (qPCR) with a TaqMan Gene Expression Assay and ABI PRISM 7900HT Sequence Detection System (Applied Biosystems, Foster City, CA, USA). Primers and probes were purchased from Life Technology (Carlsbad, CA, USA). Human glyceraldehyde-3-phosphate dehydrogenase (GAPDH) or β-actin was used as internal controls, and measurement of the threshold cycle (Ct) was performed in triplicate. Data were collected and analyzed with the Sequence Detector Software version 2.2 (Applied Biosystems) and the standard curve method. Relative gene expression was calculated as the ratio of the genes to the internal control. Group differences were compared with Student’s *t*-test.

#### Human primer sequences

JAG1-F: 5′-AGAGGCGGCCTCTGAAGAAC-3′

JAG1-R: 5′-AGCTCAGCAAGGGAACAAGG-3′

JAG2-F: 5′-TACCAACGACTGCAACCCTCT-3′

JAG2-R: 5′-TCAACACAGATGCCACCATTGT-3′

NOTCH1-F: 5′-CTGTGTCTGCCGACGCAC-3′

NOTCH1-R: 5′-CTCGGTTCCGGATCAGGAT-3′

NOTCH3-F: 5′-CAATAAGGACATGCAGGATAGCAA-3′

NOTCH3-R: 5′-GGCGGCCAGGAATAGGG-3′

NOTCH4-F: 5′-CGGAGCCGATAAAGATGCC-3′

NOTCH4-R: 5′-AGGAATAGCGGCGTCTGCT-3′

GAPDH-F: 5′-ACCACTTTGTCAAGCTCATTTCC-3′

GAPDH-R: 5′-TGCTGTAGCCAAATTCGTTGTC-3′

β−actin-F: 5′-GCACCCAGCACAATGAAGATC-3′

β−actin-R: 5′-GGAGTACTTGCGCTCAGGAGG-3′

### Mouse model study

#### Notch1^+/−^ mice

B6.129-*Notch1*^*tm1Con*^/J colony founders were purchased from the Jackson Laboratory (stock 002797). We generated the experimental mice by breeding heterozygous mice with C57BL/6J mice and followed the genotyping protocol recommended by the Jackson Laboratory. The Institutional Animal Care and Use Committee at Johns Hopkins University approved all protocols involving mice that were used in this study.

#### Behavioral testing

Behavioral testing was performed under regular lighting starting at ~postnatal day 70 (P70) from least to most stressful, beginning with locomotion in the open field (for 1 h), then the three chamber social interaction test, and lastly prepulse inhibition (74, 78, 82, 86, 90 dB) of the startle response (120 dB), with ~1 week between tests to reduce inter-trial interference as previously described [27]. Elevated plus maze was tested on a separate cohort of mice using a standard protocol of recording number of entries and time spent in the open vs. closed arms of the maze over 5 min. For the three chamber social interaction test, experimenters blind to the genotypes recorded sniffing time [28]. The novel object recognition test was performed on a third cohort as previously described [29] with 1 h between exposure to two identical objects and testing with one object replaced by a novel object. Habituation/dishabituation to olfactory odors was performed as published [30] using cotton swabs swiped in two different male stranger cages (#1, #2). The tested mouse was exposed to social odor #1 three times followed by three exposures to social odor #2, each exposure for 2 min. The duration of sniffing the cotton tip was recorded. The social interaction test and novel object recognition test were analyzed with a two-way repeated measures analysis of variance (rmANOVA). The other behavioral tests were analyzed with a Student’s *t*-test. The sample size was determined based on our previous experience with other mouse models.

#### Rapamycin treatment

Mice were treated with rapamycin modifying a published protocol [31]. In short, we injected rapamycin dissolved in DMSO (5 mg/kg, i.p.) after the habituation session over the 3 days preceding the social interaction test. We did not use a randomization method to determine how mice were allocated to experimental groups.

### Correlation between the expression of Notch–JAG pathway molecules and clinical phenotypes in first episode psychosis (FEP) patients

#### The FEP cohort

The study with the FEP cohort was approved by the Johns Hopkins School of Medicine Institutional Review Board and performed in accordance with the Code of Ethics of the World Medical Association. We obtained written informed consent from adult participants aged 18 years or older. FEP patients were enrolled within 24 months after onset. Clinical diagnosis was made by board certified psychiatrists (NGC, FCN, JMC, and TWS) based on the DSM-IV [26], and the recruitment chart was also reviewed by other psychiatrists (GN and AS). Through this team approach, we constantly monitored the inter-rater reliability in any assessment. The patients were recruited from outpatient and inpatient units in the Johns Hopkins Schizophrenia Center. Healthy controls were recruited from the general population through flyers posted at Johns Hopkins Medicine and an ad hoc advertisement in a local magazine. Further information about the recruitment and eligibility criteria for this overall cohort can be found in previous papers [32–43]. Given the possibility that tobacco and cannabis use may change the expression profiles in olfactory neuronal cells, we used 30 FEP patients and 48 healthy controls who self-reported no tobacco or cannabis use for the present study. Both clinical data and molecular expression data from olfactory neuronal cells (measured by bulk RNA-Seq) were available from all these subjects (Supplementary Table 1). However, genome sequencing information is not available for this cohort, which is a limitation.

#### Assessment of positive and negative symptoms

Patients completed the Scale for the Assessment of Negative Symptoms (SANS) [44] and Positive Symptoms (SAPS) [45]. Study clinicians (NC, FCN, JMC, TWS) performed the assessments.

#### Assessment of general neurocognition

Patients completed a two-hour battery of neuropsychological tests to assess neurocognitive function. Composite scores based on five dimensions, i.e., processing speed, verbal memory, visuospatial memory, ideational fluency, and executive function were used for the analyses [37, 46].

#### Assessment of emotional face processing in social cognition

Patients underwent a facial affect recognition task and face memory task with the Karolinska Directed Emotional Faces (KDEF) displaying happy, angry, sad, or neutral faces [47]. In the first task, facial affect recognition, participants were instructed to identify the correct expression out of the four options. In the second task, face memory, the participants had to identify whether each face had already appeared in the first task. Accuracy and response time were evaluated for each task, i.e., four domains were addressed: recognition accuracy, recognition response time, memory accuracy, and memory response time. See the methodological details in the past publications [39, 40].

#### Bulk RNA-Seq data for the expression study of the FEP cohort

Total RNA was isolated from olfactory neuronal cells using the RNeasy Plus Mini Kit (Qiagen). RNA quality was assessed on the Agilent Fragment Analyzer using a RNA High Sensitivity kit (DNF-472) and quantified using a Qubit 4 RNA BR kit (Thermo Fisher). RNA libraries were prepared with 500 ng total RNA. Library generation was accomplished using the NEBNext Ultra II Directional RNA Library Prep Kit for Illumina (E7760 and E7490) following the NEBNext Poly(A) mRNA Magnetic Isolation Module protocol. Libraries were enriched using 11 cycles of PCR amplification. Library quality and quantification were assessed on the Agilent Fragment Analyzer using a High Sensitivity NGS Kit (DNF-474) and a Qubit 4 RNA BR kit (Thermo Fisher). Samples were then normalized to 4 nM and pooled in equimolar amounts. Paired-End Sequencing was performed using Illumina’s NovaSeq6000 S4 200 cycle kit.

FastQC was used to check the quality of reads [48]. High quality data were obtained from raw data by using cutadapt to remove adapters, primers, and reads with low quality (option -q 10) or shorter than 20 nt [49]. Hisat2 (option --dta) was used to map the clean reads to the human genome, version GRCh38 (Genome Reference Consortium Human Build 38). Stringtie was used to assemble and merge transcripts and estimate transcript abundance [50]. A Python script (prepDE.py) provided by the Stringtie developer was used to create count tables for differential expression analysis. Principle component analysis was conducted to control the quality of the data, and no outlier was detected (Supplementary Fig. 1). Fragments Per Kilobase of transcript per Million mapped reads (FPKM) were calculated to quantify the expression levels of genes for downstream analysis.

#### BrainSeq database

The BrainSeq database has been developed by Lieber Institute, which provides expression data and eQTL data (http://eqtl.brainseq.org). These were based on the RiboZero RNA-seq data in the dorsolateral prefrontal cortex from 286 SZ patients and 265 healthy controls, and seminal publications were made with this dataset [51, 52].

#### Statistical analyses

Statistical analyses for clinical data were conducted by using STATA 15 and R version 3.5.3.

Multivariable regression analyses were performed to examine the correlation between the expression (FPKM) of *NOTCH1/JAG1* and clinical test scores (positive and negative symptoms, neurocognition, and social cognition) collected from the FEP cohort. Analyses were conducted for males and females separately. Age, race, and diagnosis (control or patient) were controlled for the analysis for the pooled group (including both FEP patients and controls); age and race were controlled for the analysis for the control group; while age, race, chlorpromazine equivalent dose estimated by the Defined Daily Doses method [53], and duration of illness were controlled for the analysis for the patient group. A permutation test was performed to evaluate statistical significance.

Multivariable regression analyses were also performed to examine a possible difference between males and females in variables (e.g., *JAG1* expression and memory accuracy). In the analyses, we defined *JAG1* expression or memory accuracy as a dependent variable; sex as an independent variable; as well as age, race, and diagnosis (for the pooled group), age and race (for the control group), and age, race, chlorpromazine equivalent dose, and duration of illness (for the patient group), as covariates.

## Results and discussion

### Downregulation of Notch–JAG pathway molecules in olfactory neurons from patients with SZ compared with healthy controls

We first collected microarray data of laser-captured olfactory neurons and compared gene expression profiles between patients with chronic SZ and healthy subjects. We previously analyzed this dataset for a different scientific aim [15]. Intriguingly, the Notch ligand *JAG1* was a top hit (the significance ranking was sixth out of 22,574 differentially expressed genes), which was significant after multiple testing correction (FDR < 0.05) (Table 1). Different probes for *JAG1* consistently indicated downregulation in SZ patients. In the Dll ligand family, only *DLL1* was detected and was not changed in the microarray analysis. In addition to the significant downregulation of *JAG1*, we also observed lower expression of *JAG2, NOTCH1*, *NOTCH3*, and *NOTCH4* (with no change for *NOTCH2*) in SZ (Table 1). However, they did not survive multiple testing correction. Thus, we aimed to confirm these observations for *JAG1*, *JAG2*, *NOTCH1*, *NOTCH3*, and *NOTCH4* by qPCR. We observed consistent downregulation in all the molecules tested when we normalized their expression with either β-actin or GAPDH (Table 1). Among them, downregulation of *NOTCH1* was the most robust (70% downregulation) and statistically significant (microarray: *p*-value = 0.02; qPCR β-actin normalization: *p*-value = 9.90E−03; and qPCR GAPDH normalization: *p*-value = 0.02) (Table 1). We also looked for publicly available gene expression data from postmortem brain collection from SZ patients and controls (the BrainSeq database). We found the downregulation of *NOTCH1* (*p*-value = 0.0014) and *JAG1* (*p*-value = 0.011) in the dorsolateral prefrontal cortex, but these were not FDR-significant. There were significant correlations between SNPs (*NOTCH1* and *JAG1*) and their expression levels: 94 SNPs of *NOTCH1* and 291 SNPs of *JAG1* were significantly correlated with expression levels after multiple comparison correction. However, these SNPs did not reach the significant levels in the GWASs for SZ. Altogether, we hypothesized that the Notch–JAG pathway, through expression changes, may be associated with at least some specific pathological dimensions underlying SZ.

**Table 1:** Microarray and quantitative real time-PCR results of the Notch–JAG pathway gene expression in human olfactory neural epithelium from healthy controls (HC) and chronic schizophrenia patients (SZ).

Gene	Microarray		RT-PCR									
			β-actin normalization					GAPDH normalization				
			Mean			*t*-test		Mean			*t*-test	
	Fold	*p*-value	HC	SZ	SZ/HC	*p*-value	Sig	HC	SZ	SZ/HC	*p*-value	Sig
JAG1	0.82	2.77E−06	8.5	6.3	0.74	0.19	ns	7.1	5.5	0.78	0.34	ns
JAG2	0.74	0.03	29.6	17.6	0.59	4.63E−02	*	26.6	16.4	0.62	0.1	ns
NOTCH1	0.7	0.02	3.9	1.2	0.31	9.90E−03	**	3.3	1.1	0.34	0.02	*
NOTCH3	0.81	0.03	195.7	100.3	0.51	0.05	ns	178.4	98.9	0.55	0.1	ns
NOTCH4	0.64	9.39E−03	53	27.6	0.52	0.06	ns	43.1	22.8	0.53	0.1	ns

Significant results (Sig) are highlighted as follows: *p < 0.05; **p < 0.001.

*ns* not significant.

### Behavioral deficits in *Notch1* heterozygote knockout mice

Only a few studies have addressed the influence of the Notch–JAG pathway on higher brain function in adulthood and, as far as we are aware, comprehensive studies in this context are limited to *Notch1* genetic models mainly for learning and memory [54]. *Notch1* homozygote knockout mice are embryonically lethal, but heterozygous knockout mice survive to adulthood [55]. Furthermore, as described above, our expression study of olfactory neurons found *NOTCH1* to be significantly and robustly downregulated in SZ patients compared with healthy controls. Thus, we decided to use *Notch1* heterozygote knockout (*Notch1*^*+/*−^) mice to shed light on the functional implication of the overall Notch1 pathway in higher brain function in adulthood.

**Fig. 1:**
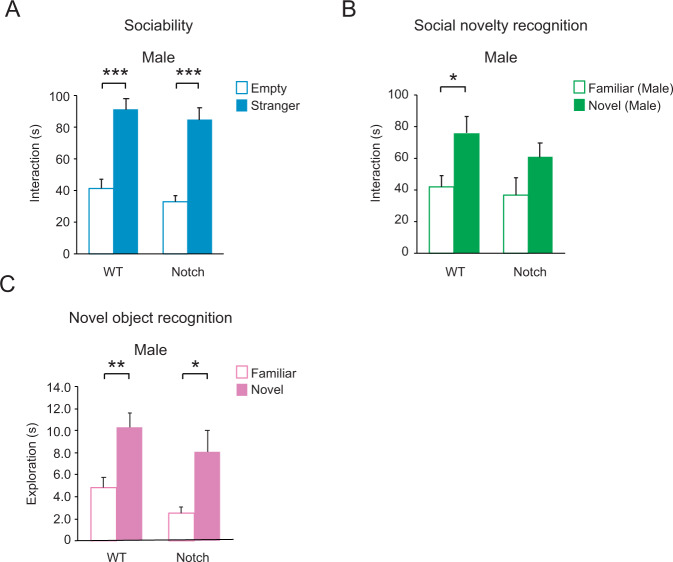
Selective behavioral deficits of *Notch1*^*+/*−^ males only in social novelty recognition, but not in sociability. **A** *Notch1*^+/−^ males behaved normally in the sociability phase of the test, preferring to interact with a stranger male (solid bar). WT *n* = 8, Notch *n* = 9 **B** *Notch1*^*+/−*^ males did not show a significant preference for a novel male mouse (solid bar) over a familiar male mouse (open bar) in the social novelty recognition test. WT *n* = 8, Notch *n* = 9 **C** *Notch1*^*+/*−^ males showed normal novel object recognition 1 h after first exposure. Both WT and Notch mice display a normal preference for a novel object (solid bars). WT *n* = 12, Notch *n* = 10 Two-way repeated measures analysis of variance (rmANOVA) with Bonferroni adjusted post-hoc analysis. Data are shown as mean ± SEM. *p < 0.05, **p < 0.01, and ***p < 0.001.

Contrary to our expectation, *Notch1*^*+/*−^ mice displayed almost no abnormality in several representative dimensions for higher brain function. We did not observe any deficits in the open field, prepulse inhibition, elevated plus maze, and novel object recognition (Table 2). In contrast, they displayed deficits only in social novelty recognition in the three chamber social interaction test. In this test, *Notch1*^*+/*−^ mice showed normal sociability (that is, preference for a stranger over the empty side) (Fig. 1A), but were impaired in social novelty recognition, not displaying a preference to a novel mouse over a familiar mouse (Fig. 1B). *Notch1*^*+/*−^ mice showed normal novel object recognition (Fig. 1C), so we can conclude that the novelty recognition deficit is specific to a social context.

**Table 2:** Selective behavioral deficits of *Notch1*^*+/*−^ males only in a social novelty recognition behavior, but not in other behavioral paradigms.

RDoC domain construct	Behavior	Test	Result
Arousal/Regulatory systems Arousal	Locomotion	Open field	No difference
Sensorimotor systems	Sensorimotor gating	Prepulse inhibition	
Negative valence Potential threat (anxiety)	Anxiety-like	Open field	
		Elevated plus maze	
Cognitive systems	Object novelty recognition	Novel object recognition	
Systems for social processes	Sociability	Three chamber social interaction	
	**Social novelty recognition**		**Impaired**

Significant results are highlighted in bold.

### Male-specific deficits of social novelty recognition in *Notch1* heterozygote knockout mice

**Fig. 2:**
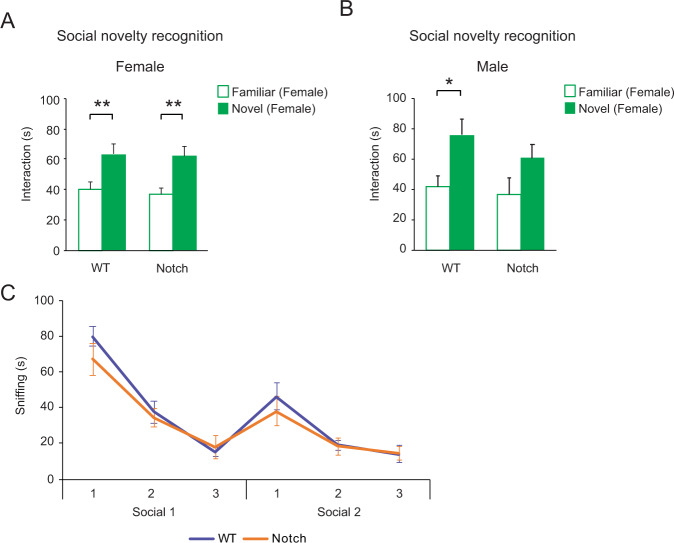
Sex specific deficit of *Notch1*^*+/*−^ mice in a social novelty recognition behavior. **A** *Notch1* ^+/−^ females display normal preference for novel female strangers (solid bar) in the social novelty recognition test. WT *n* = 12, Notch *n* = 16 **B** *Notch1*^+/−^ males are also impaired in social recognition towards female strangers (solid bar). WT *n* = 20, Notch *n* = 13 **C** *Notch1*^*+/*−^ males showed normal habituation and dishabituation to social odors. There was no difference between WT (purple) and Notch (orange) in any of the 6 trials. WT *n* = 10, Notch *n* = 8. Two-way repeated measures analysis of variance (rmANOVA) with Bonferroni adjusted post-hoc analysis. Data are shown as mean ± SEM. *p < 0.05 and **p < 0.01.

Intriguingly, the social novelty recognition deficit was sex-specific; *Notch1*^*+/−*^ females showed normal preference for a stranger mouse (Fig. 2A). On the other hand, *Notch1*^*+/*−^ males were impaired not only in recognizing novel males (the standard test shown in Fig. 1B), but also when tested unconventionally against novel females (Fig. 2B). *Notch1*^*+/−*^ males showed normal habituation and dishabituation to social odors, so the social novelty recognition deficit is not due to an olfactory impairment (Fig. 2C), a critical experiment given the involvement of the Notch–JAG pathway in the development and function of the olfactory system [56, 57]. Based on this result, one copy of *Notch1* is sufficient for a normal response to social odors. Our finding of normal olfaction in *Notch1*^*+/−*^ male mice may not contradict our expression data showing *NOTCH1* downregulation in human olfactory neurons since they are used as a surrogate tissue from living patients to estimate molecular signatures of neurons in the brain. Inspired by the beneficial effects of rapamycin in mouse models that show deficits in social behaviors, we treated the *Notch1*^+/−^ males with rapamycin (5 mg/kg) over the 3 days of habituation preceding the social interaction test [31]. After treatment with rapamycin, the social novelty recognition deficit in *Notch1*^+/−^ male mice was ameliorated (Supplementary Fig. 2).

### Male-specific deficits in social cognition in FEP patients

Given that only male *Notch1*^*+/*−^ mice displayed a social novelty recognition deficit, we hypothesized that the Notch-JAG pathway may have a specific role in social cognition only in male humans. To address this question, we studied how the *JAG1* or *NOTCH1* expression level may correlate with higher brain function in living patients. Unfortunately, we did not conduct deep phenotyping for the cohort of chronic SZ patients and healthy controls described above, from which we had obtained the expression data on laser-captured olfactory neurons. However, we have established another cohort in which FEP patients and healthy controls are characterized with deep phenotyping (symptomatic assessment, neurocognition, and social cognition) and olfactory neuronal cells enriched from nasal tissue (Supplementary Table 1) [32–43]. These olfactory neuronal cells have also been used as a surrogate tissue to estimate neuronal signatures by multiple groups, including ours [23, 58–60]. We collected bulk RNA-Seq data from olfactory neuronal cells and calculated FPKM values to assess the expression levels of *NOTCH1* and *JAG1*.

We first assessed whether the expression level of *NOTCH1* or *JAG1* was different between FEP patients and healthy controls; however, the expression levels were not significantly different. Although the data are different from the chronic SZ cohort, these results are within our anticipation. It is known that some biological measures will show differences between chronic SZ patients and controls but may look similar between FEP patients and matched controls [61–63]. In these cases, investigators have speculated that the reason for not detecting a significant difference between FEP patients and controls may be because some pathological signatures are not strong enough during early stages of the disease. Even in such cases, a correlation between phenotypic and molecular changes at the pre-symptomatic level can be expected.

**Fig. 3:**
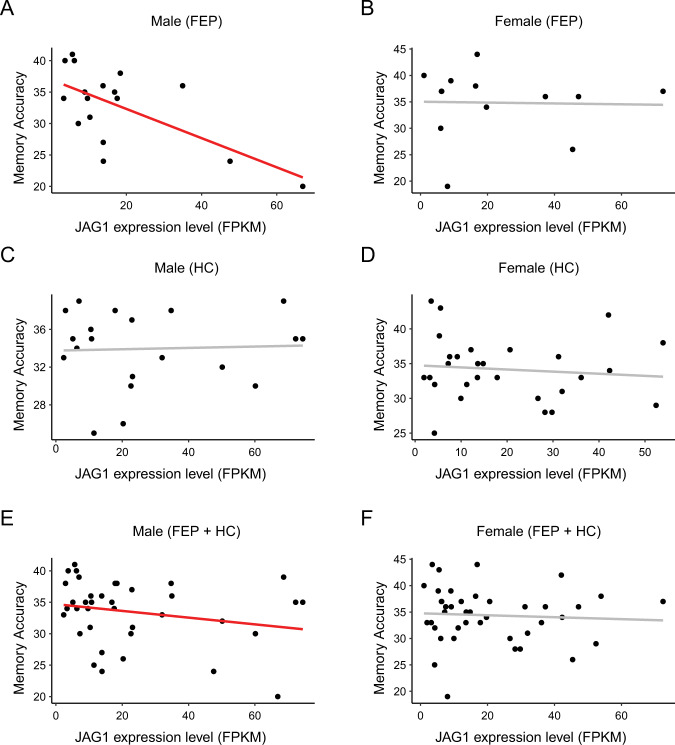
Correlations between *JAG1* expression and memory accuracy in the FEP cohort. **A** There was a significant correlation observed in male FEP patients. **B** There was no significant correlation observed in female FEP patients. **C** There was no significant correlation observed in male healthy controls (HC). **D** There was no significant correlation observed in female HC. **E** There was a significant correlation observed in male subjects (combining both FEP patients and HC). **F** There was no significant correlation observed in female subjects (combining both FEP patients and HC) Black dots represent individual subjects. A red line represents a significant correlation, while a gray line represents a correlation that did not reach the significance cutoff.

Thus, we tested the correlation between gene expression in olfactory neuronal cells and multiple clinical/neuropsychological scales. These include the SAPS [45], SANS [44], general neurocognition using a composite score based on five dimensions, i.e., processing speed, verbal memory, visuospatial memory, ideational fluency, and executive function [32, 33, 37, 46], and emotional face processing in social cognition [39, 40, 47]. We did not observe a correlation between the molecular expression (*JAG1* or *NOTCH1*) and symptomatic changes as assessed by the SAPS and SANS or the scales of general neurocognition (Supplementary Table 2). However, in a facial affect recognition task and face memory task with the KDEF [47], in which recognition accuracy, recognition response time, memory accuracy, and memory response time were measured, we observed a negative correlation between *JAG1* expression and memory accuracy in male FEP patients (*p*-value = 0.03) (Table 3). This correlation was not observed in female FEP patients, in male healthy controls, or in female healthy controls The correlation between *JAG1* expression and memory accuracy remained significant in males (*p*-value = 0.04) when combining FEP patients and healthy controls, but not in females (Fig. 3). Note that there were no significant sex differences in either *JAG1* expression or memory accuracy (Supplementary Table 3).

**Table 3:** Correlation of *JAG1* and *NOTCH1* expression with emotional face processing measures in the FEP cohort.

Group	Sex	Gene	Clinical variable	Correlation coefficient	*p*-value
FEP	Male	NOTCH1	Memory accuracy	−0.395	0.293
			Memory response time	−0.134	0.752
			Recognition accuracy	−0.019	0.960
			Recognition response time	−0.083	0.831
		JAG1	**Memory accuracy**	**−0.716**	**0.030**
			Memory response time	0.686	0.060
			Recognition accuracy	−0.563	0.114
			Recognition response time	0.664	0.051
FEP	Female	NOTCH1	Memory accuracy	−0.057	0.904
			Memory response time	−0.670	0.100
			Recognition accuracy	−0.239	0.606
			Recognition response time	−0.702	0.079
		JAG1	Memory accuracy	0.019	0.968
			Memory response time	−0.609	0.147
			Recognition accuracy	0.390	0.388
			Recognition response time	−0.314	0.493
HC	Male	NOTCH1	Memory accuracy	−0.016	0.949
			Memory response time	−0.234	0.350
			Recognition accuracy	0.040	0.875
			Recognition response time	−0.313	0.207
		JAG1	Memory accuracy	0.141	0.578
			Memory response time	0.382	0.118
			Recognition accuracy	0.060	0.813
			Recognition response time	−0.183	0.468
HC	Female	NOTCH1	Memory accuracy	0.136	0.509
			Memory response time	−0.122	0.554
			Recognition accuracy	−0.051	0.805
			Recognition response time	0.277	0.171
		JAG1	Memory accuracy	−0.152	0.460
			Memory response time	−0.154	0.452
			Recognition accuracy	0.348	0.082
			Recognition response time	−0.167	0.415

Significant results are highlighted in bold (*p* < 0.05).

*FEP* first episode psychosis, *HC* healthy controls.

### The significance of the present study: from a newly generated hypothesis through mouse and human studies to further validation

By uniquely and effectively combining studies from both human and mouse models we now propose a new working hypothesis that the Notch–JAG pathway may be specifically involved in a specific dimension of social cognition in a sex-dependent manner. In this exploratory and hypothesis-generating work, the mechanistic dissection of this hypothesis is beyond the scope. However, we wish to emphasize the involvement of the Notch–JAG pathway molecules specifically in the dimension of social cognition, but not in many other symptomatic or cognitive domains in both human and mouse studies. Furthermore, in both mouse and human studies the association with Notch1 existed only for the social memory phase of the test, and not for the more affective phase of the test. A mouse model of *Notch1* haploinsufficiency may be a good tool to address the potential mechanism.

Mice in which *Notch1* has been conditionally deleted by crossing with the αCaMKII-Cre line also show a social novelty recognition deficit (no information on sex) [64], suggesting that deletion of Notch1 from forebrain pyramidal neurons postnatally is sufficient to impair this behavior. This also implies that the deficit is not due to effects on early development. We showed that a short rapamycin treatment before the social interaction test could ameliorate the social novelty recognition deficit, again implying a role for Notch1 in adulthood. Although one of pharmacological actions by rapamycin is associated with an inhibition of mTOR, the relationship between the Notch and mTOR pathways has been reportedly complex and context-dependent. Instead of extending superficial speculations, we take the experimental results with rapamycin as a proof that adult behavioral deficits elicited by *Notch1* haploinsufficiency are treatable by an intervention in the adult stage.

Nevertheless, a mechanistic involvement of alterations in the Notch–JAG pathway for male-specific social cognition remains an important question for future investigations. There have been some biological assessments of the Notch–JAG pathway in patients with neuropsychiatric disorders. For example, attenuated Notch signaling has been reported based on measures in plasma in SZ and bipolar disorder [65]. An abnormal expression pattern of NOTCH-related genes, including reduced *JAG1* expression, was also reported in the dorsolateral prefrontal cortex and amygdala of suicide victims [66]. Clinical studies, particularly those that directly explore the pathological regions in the brain, will be useful when combined with animal studies in which a region-specific knockout approach for *Notch1* and related molecules are employed. Through these translational studies, we may be able to define important brain regions and circuitry associated with the Notch–JAG pathway in social cognition in a male-specific manner.

We have to acknowledge that there are some gaps between the data from the chronic SZ cohort and those from the FEP cohort, although we could extract an important common message from them. As we described above, some differences are related to the levels of disease progression. In general, chronic patients with SZ have a higher accumulative dose of antipsychotics, which may affect gene expression to some extent. Age difference and other factors may also be involved. These are potential limitations of the present study.

Biopsied cells from living subjects enable almost real-time correlation studies of molecular profiles with clinical phenotypes in the same individual, avoiding the many confounds of postmortem tissue [15, 16, 60, 67]. In this study, we used olfactory neuronal cells that are easily and safely accessible in living subjects, and nevertheless represent neuronal molecular signatures to a reasonable extent [23, 68]. We acknowledge that the olfactory resource may not fully address region specific and neuron-subtype specific questions associated with the brain. Therefore, biopsied tissue and postmortem brain have complementary significance. We also believe that studying molecular expression in biopsied tissues relevant to neuronal signatures is complementary to GWAS studies, in particular when they are moving in the direction of looking at the genomic impact on specific behavioral constructs [69].

## Supplementary information

Supplemental material
