# Supplementary material for: Sex-specific involvement of the Notch–JAG pathway in social recognition
Source: Transl Psychiatry. 2022 Mar 10;12:99. doi: 10.1038/s41398-022-01867-4 (PMC8913639; doi:10.1038/s41398-022-01867-4)
Supplement: Supplementary file 1 — Supplemental material [file 41398_2022_1867_MOESM1_ESM.pdf]

Table S1. Demographic summary of study participants in the FEP cohort.

Characteristic	Mean (±SD)		p-value
	FEP (*n* = 30)	HC (*n* = 48)	
Age (years)	23.867 ± 4.516	23.833 ± 3.485	0.973
Sex			
Male	17	20	0.246
Female	13	28	
Race			
African American	19	31	0.823
Caucasian	9	12	
Other	2	5	
CPZ dose (mg)	261.354 ± 226.282	N/A	
DOI (month)	14.867 ± 9.971	N/A	

Only subjects who self-reported no tobacco or cannabis use were included in the present study. SD: standard deviation; FEP: first episode psychosis; HC: healthy controls; CPZ: Chlorpromazine; DOI: duration of illness

Table S2. Correlation between *JAG1* and *NOTCH1* expression and clinical variables in the FEP cohort.

Group	Gender	Gene	Clinical variable	Correlation coefficient	p-value
FEP	Male	NOTCH1	NP composite score	0.469	0.124
			SANS total	0.154	0.652
			SAPS total	0.070	0.837
		JAG1	NP composite score	-0.359	0.252
			SANS total	0.425	0.193
			SAPS total	0.115	0.735
FEP	Female	NOTCH1	NP composite score	0.298	0.516
			SANS total	0.401	0.373
			SAPS total	-0.083	0.860
		JAG1	NP composite score	0.498	0.255
			SANS total	-0.654	0.111
			SAPS total	-0.053	0.910
HC	Male	NOTCH1	NP composite score	0.018	0.932
			SANS total	N/A	N/A
			SAPS total	N/A	N/A
		JAG1	NP composite score	0.174	0.407
			SANS total	N/A	N/A
			SAPS total	N/A	N/A
HC	Female	NOTCH1	NP composite score	0.067	0.731
			SANS total	N/A	N/A
			SAPS total	N/A	N/A
		JAG1	NP composite score	-0.186	0.333
			SANS total	N/A	N/A
			SAPS total	N/A	N/A

FEP: first episode psychosis; HC: healthy controls; NP: Neuropsychological test; SANS: Scale for the Assessment of Negative Symptoms; SAPS: Scale for the Assessment of positive Symptoms

Table S3. Group comparison between males and females in the FEP cohort (p-value).

Variables	FEP	HC	FEP + HC
Memory accuracy	0.2	0.662	0.384
JAG1 expression	0.316	0.122	0.525

FEP: first episode psychosis; HC: healthy controls

Supplementary figure 1

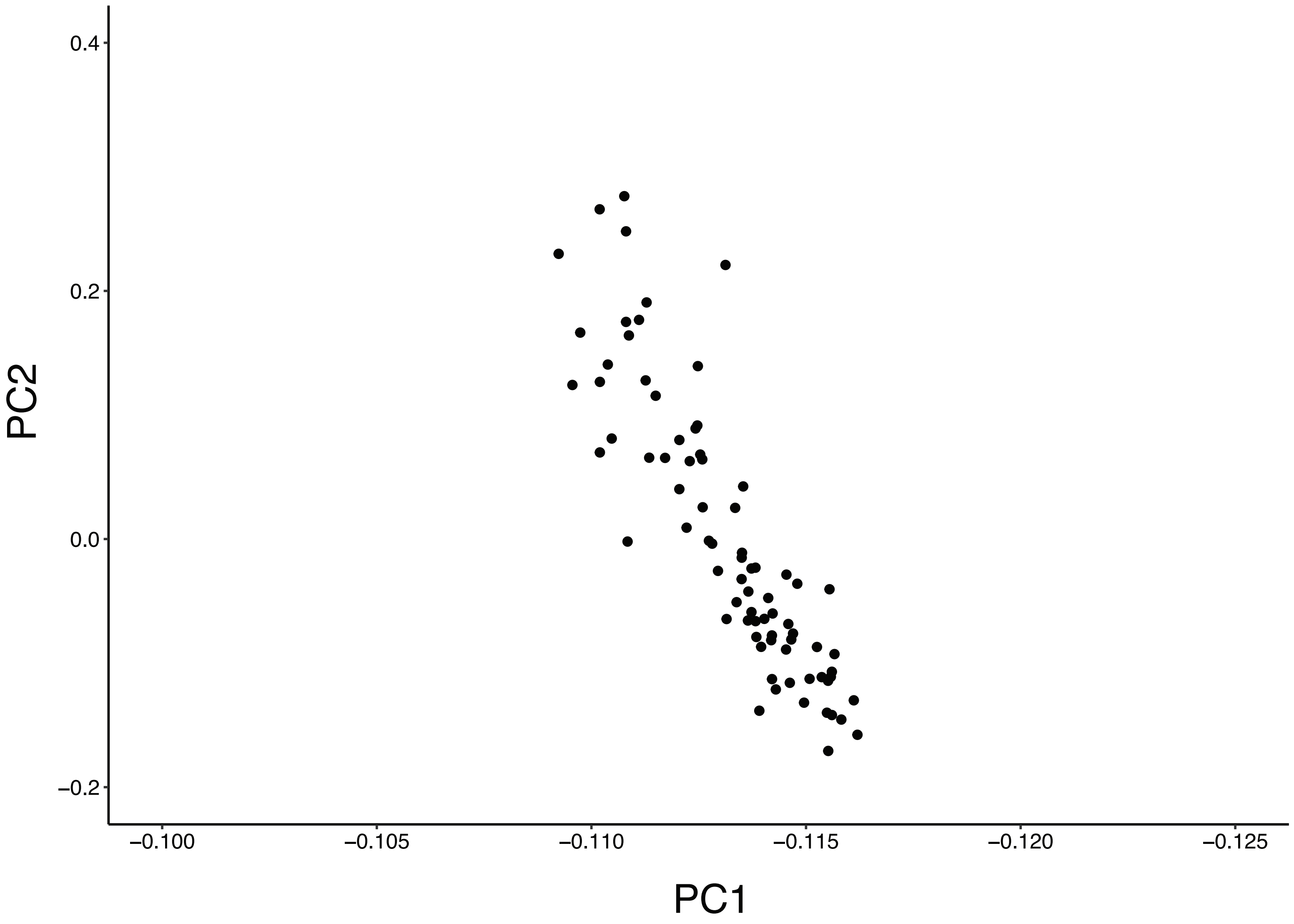

Supplementary Fig. 1 Principal component analysis (PCA) of bulk RNA-Seq data from FEP cohort. We conducted PCA to address the quality of the RNA-Seq data. No outlier was identified by this analysis.

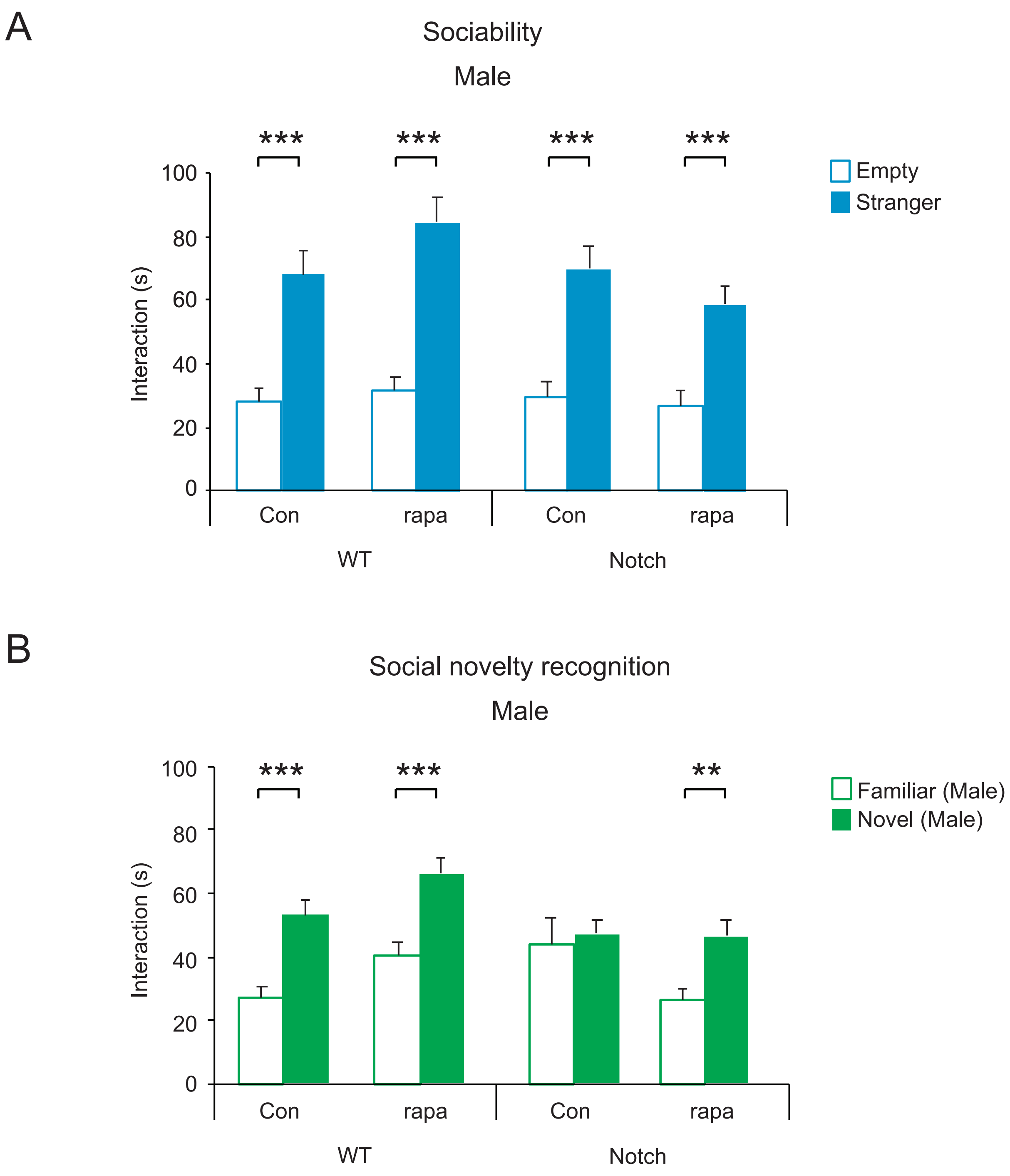

Supplementary Fig. 2 Rapamycin ameliorates the social novelty recognition deficits in *Notch1*$^{+/-}$ males.
Mice were injected i.p. with DMSO vehicle (Con) or 5 mg/kg of the mTORC1 inhibitor rapamycin (rapa) once a day over the 3 days of habituation preceding the three chamber social interaction test.
A All 4 groups preferred the stranger (solid bars) and rapamycin had no effect on the sociability phase of the test. n = 11-17
B The social novelty recognition deficit in Notch males was rescued by rapamycin treatment. n = 11-17
Two-way repeated measures analysis of variance (rmANOVA) with Bonferroni adjusted post-hoc analysis. Data are shown as mean ± SEM. **p<0.01 and ***p<0.001
